# Allicin modulates the intestinal microbiota to attenuate blood glucose and systemic inflammation in type 2 diabetic rats

**DOI:** 10.3389/frmbi.2023.1102694

**Published:** 2023-04-25

**Authors:** LinZehao Li, Yan Yan, Xiaolei Wang, Yanli Hou, Lina Ding, Zhibin Wang, Qinghe Song, Wenyu Ding, Xiandang Zhang

**Affiliations:** ^1^ Endocrine and Metabolic Diseases Hospital of Shandong First Medical University, Shandong First Medical University & Shandong Academy of Medical Sciences, Jinan, China; ^2^ Department of Surgery, Qilu Hospital of Shandong University, Jinan, China; ^3^ Department of Pharmacy, Linyi People’s Hospital, Linyi, China

**Keywords:** type 2 diabetes mellitus, intestinal microbiome, allicin, prebiotics, inflammation, short-chain fatty acids

## Abstract

**Introduction:**

Allicin is a wide spectrum prebiotic for human health, but whether it can attenuate blood in diabetes patients is rarely reported. In this study, we built a rat model and investigated the effect of allicin on diabetes mellitus type 2 (T2DM). We found that allicin could effectively reduce blood glucose levels, regulate intestinal microbiota, reduce lipid and body weight accumulation, and systemic inflammation in T2DM rats.

**Methods:**

The rat model of type 2 diabetes was made by streptozotocin, and different doses of allicin were given orally by gavage. The intestinal contents of diabetes rats were sequenced and analyzed by 16S technology, and the clinical indicators of rats were detected for joint analysis.

**Results:**

Allicin can improve the intestinal flora of type 2 diabetes rats, enrich beneficial metabolites, reduce blood glucose, improve blood lipids, reduce systemic inflammation, and improve type 2 diabetes.

**Discussion:**

Intestinal microbiome analysis showed that allicin gavage significantly regulated the structure and main components of the intestinal microbiota in T2DM rats. Allicin increased the abundance of probiotic microbes, such as *Lactobacillus*, *Clostridium* and *Akkermansia*, while it reduced pathogenic microbes, such as Enterobacter, Erysipelatoclostridium and Colidextribacter. Allicin gavage increased the abundance of intestinal short-chain fatty acids, such as acetic acid and propionic acid. Correlation analysis showed that the increased gut microbes by allicin gavage were significantly associated with health physiological parameters but negatively related to serum inflammatory factors such as interleukin-6 (IL-6), tumor necrosis factor alpha (TNF-a), and hypersensitive C-reactive protein (hs-CRP). In addition, our study also suggests that allicin may have prebiotic effects on chronic liver injury. This study shows that allicin can regulate various clinical symptoms of T2DM and is a potential therapeutic drug for T2DM.

## 1 Introduction

Type 2 diabetes mellitus (T2DM) has now become one of the main chronic metabolic diseases worldwide, and epidemiological data reveal an unstoppable upward trend over the next ten years (Ogurtsova et al., 2017). According to estimates from the International Diabetes Federation (IDF), 5 million people died from diabetes in 2017, and in 2019, there were approximately 463 million people with diabetes worldwide, a number that will rise to 578 million by 2030 (Saeedi et al., 2019). Complications from T2DM can also lead to serious diseases, such as retinopathy due to hyperglycemia, diabetic foot and diabetic nephropathy (Nathan, 1993), which put tremendous stress on society and public health care (Forouhi and Wareham, 2014). The WHO Global Report on Diabetes showed that the loss of GDP from diabetes, including the direct and indirect costs of diabetes, is $1.7 trillion each year, including $800 billion in low- and middle-income countries (World Health Organization, 2016). At present, drugs are helpful in alleviating the blood glucose disturbance of T2DM, but it is difficult to completely cure T2DM. At the same time, taking certain T2DM drugs for a long period may have certain side effects on the human body. Therefore, developing new, affordable, safe and effective treatment strategies has become a top priority.

Recent studies have identified the intestinal microbiota as a potential target for the treatment of T2DM (Blandino et al., 2016). First, the probiotic intestinal microbiota can repair the function of the intestinal barrier and prevent the release of endotoxins into the blood (Everard and Cani, 2013). Second, they can help us to decompose dietary fiber and produce short-chain fatty acids (SCFAs), and an increase in SCFAs in the human body could help to further alleviate T2DM (Kim, 2018). In addition, the gut microbiota modulates glucagon-1 (GLP-1), peptide YY (PYY), glucose-dependent insulinotropic peptide (GIP) and other markers in T2DM patients and regulates energy metabolism through the gut-brain axis to ameliorate the side effects induced by high blood glucose (Asadi et al., 2022).

Studies have shown that the intestinal microbiota is an expected target for the treatment of T2DM (Larsen et al., 2010; Burcelin et al., 2011; Gurung et al., 2020). There is a growing trend of diabetic drug research targeting the intestinal microbiota, such as flavopiridol (Pang et al., 2015), inulin (Zhang Q. et al., 2018), and chitosan (Prajapati et al., 2015). Clinical trials have also demonstrated that the transplantation of healthy human fecal microbiota to T2DM patients could ameliorate their clinical index (Ng et al., 2022). There are many drugs in traditional Chinese medicine that can regulate the intestinal microbiota to improve human health, which provides an endless source of ideas for the development of intestinal microbiota-targeted drugs for T2DM.

Allicin, the natural extract of garlic, mainly exists in the form of odorless alliin (S-allyl-L-cysteine sulfoxide) (Miron et al., 2004; Borlinghaus et al., 2014) and has been reported to have a wide spectrum of prebiotic effects (Choo et al., 2020; Marón et al., 2020; Panyod et al., 2020; Nadeem et al., 2021). Animal model studies have shown that allicin can modulate the intestinal microbiome in obesity (Shi et al., 2019), atherosclerosis (Wu et al., 2015), etc. However, it is still unknown whether allicin can play a role in the treatment of T2DM and its complications. Here, we established a T2DM rat model to study the prebiotic effect of allicin on diabetes. The clinical index of the rat model was tested, their intestinal flora were analyzed, and the effect of allicin on the intestinal microbiota and clinical index was studied by Spearman correlation analysis. This study provides a new basis for the prebiotic effect of allicin on T2DM by modulating the intestinal microbiota.

## 2 Materials and methods

### 2.1 Ethics approval

This study was approved by the Endocrine and Metabolic Disease Hospital, Shandong First Medical University, 2021-005. All animal experiments were performed under the guidance of the Animal Care Committee of Shandong First Medical University and under standard operating procedures.

### 2.2 Animal experiments

SD rats were purchased from Charles River Laboratories (CRL), Beijing, China. Rats were raised in the SPF lab under a normal diet and unlimited water intake and maintained under the appropriate temperature (23°C) and light conditions (12:12-h light-dark cycle).

After an adaptive feeding cycle, the rats were administered intraperitoneal injections of 20 mg/g streptozotocin (STZ) until the T2DM rat model was established. The T2DM model rats and normal rats were divided into five groups: 1) low-fat, low-sugar-fed normal SD rats (NC group); 2) STZ-induced gavage with sodium carboxymethylcellulose (0.5%, 6 ml/kg), high-fat-fed T2DM rats (M group); 3) STZ-induced gavage with 30 mg/kg allicin, high-fat-fed T2DM rats (AL group); 4) STZ-induced gavage with 70 mg/kg allicin, high-fat-fed T2DM rats (AH group); and 5) STZ-induced gavage with 100 mg/kg metformin, high-fat-fed T2DM rats (Met group), with 5 rats in each group. The drugs were administered orally 6 times per week. Blood glucose was measured by tail vein blood collection (ACCU-CHEK, Roche, Basel, Switzerland) every Wednesday at 3 pm after 8 h of fasting. Because allicin is insoluble in water, it is miscible in sodium carboxymethyl cellulose (0.5%). Allicin was purchased from Source Leaf Biological, Shanghai, China, and metformin hydrochloride and sodium carboxymethylcellulose were purchased from Solarbio.

When STZ was used for intraperitoneal injection, a small dose of slow injection was carried out in strict accordance with the standard method, while nicotinamide was used for protection, so that the blood glucose level of the model rats after modeling was not high, but there was a significant difference with the NC group (Furman, 2015). Nicotinamide was used to reduce model islet β cell damage, which was used to simulate pre-T2DM symptoms.

All drugs were administered in sodium carboxymethylcellulose and gavaged by gastric administration according to standard operating procedures (Chen et al., 2011). Rats in the NC group and M group were gavaged with the same volume of sodium carboxymethylcellulose. During the experimental period, the body weight, blood glucose and feed intake of each rat were monitored weekly. After 10 weeks of the experimental period, all rats were anesthetized and sacrificed. At the same time, the colonic contents and serum were collected and immediately stored in liquid nitrogen and transferred to a -80°C freezer for long-term preservation. The intestinal contents were sampled on ice to ensure that the sampling process was carried out in a clean and sterile environment. The feces were quickly transferred to sterile freezing tubes.

### 2.3 Insulin tolerance test

Insulin tolerance test (ITT) experiments were performed 5 weeks after administration. Rats were fasted for 6 h, and blood was collected from the tail vein to determine fasting blood glucose levels by ACCU-CHEK (Roche, Basel, Switzerland). After intraperitoneal injection of 0.75 U/kg insulin, the blood glucose values of the rats were measured at 0, 30, 60, 90, and 120 min.

### 2.4 Intestinal microbiome sequencing

The colon contents collected in the 10th week were sent to Novogene Biotechnology Company (Shanghai, China). Intestinal microbial DNA was extracted from each sample with the CTAB kit method (CTAB, Nobleryder, China), and then PCR was used to amplify the V3-V4 region using characteristic primers with barcodes (16V34,5’-3’: 341F,CCTAYGGGRBGCASCAG;806R,GGACTACNNGGGTATCTAAT). The buffer was Phase ® High-Fidelity PCR Master Mix with GC Buffer (M0531S, New England Biolabs, USA). The enzyme was Phusion ® High-Fidelity DNA polymerase (M0530S, New England Biolabs, USA). Intestinal microbial DNA was extracted from each sample, and a TruSeq ® DNA Sample Preparation Kit (Illumina, San Diego, CA, USA) was used for library construction. Using Qubit @ 2.0 Fluorometer (Thermo Scientific) to build the library for detection and Q-PCR quantification. After the library was qualified, a NovaSeq 6000 (Illumina Novaseq6000, Illumina, San Diego, CA, USA) was used for online sequencing. (Illumina, San Diego, CA, USA). 16S rDNA sequences were used to generate a DNA library and sequenced by the Illumina NovaSeq platform according to standard operating procedures. Quality control (QC) was performed, and high-quality 16S rDNA clean data were generalized by Novogene Company.

### 2.5 Short-chain fatty acid (SCFA) analysis

Acetic acid, propionic acid, butyric acid, valeric acid and isovaleric acid were extracted and determined in each group of stool samples by GC-MS. Ethyl acetate divided into eight gradient concentrations was applied as mixed standards. Then, 25 μL of 4-methylvaleric acid was added at a final concentration of 500 μM to the standard to act as an internal standard for the GC-MS assay.

### 2.6 Serum IL-8 and TNF-α measurement

Serum TNF-α and IL-8 were quantified by a commercial kit based on horseshoe crab deformed cell extracts (Elisa kit; Cambrex BioScience, abme).

### 2.7 Blood biochemistry analysis

Serum cholesterol (TC), triglyceride (TG), high-density lipoprotein (HDL), low-density lipoprotein (LDL), serum albumin (ALB), serum alkaline phosphatase (ALP), alanine aminotransferase (ALT), hypersensitive C-reactive protein (hs-CRP), total bilirubin (TBIL), γ-glutamyltransferase (GGT), serum calcium (Ca), phosphorus (P) magnesium (Mg) and glycosylated hemoglobin (HbA1c) were measured at the Department of Endocrinology and Metabolic Diseases Hospital, Shandong First Medical University, China.

### 2.8 Bioinformatics and statistical analysis

The 16S rDNA data of the intestinal microbiome were processed by QIIME software (QIIME 2-2020.6) at Novogene Biotechnology Company. DADA2 was used for noise reduction in QIIME2 process clustering, and each amplicon sequence variable (ASV) generated was analyzed. The correlation between intestinal microbiota and clinical manifestations (Spearman rank correlation coefficient) was performed on the Wekemo Bioincloud platform (R version 3.5.3, ggplot2 package, extrafont package, grid package, ade4 package, psych package, pheatmap package, etc.), and the relevant P value was calibrated by the Bonferroni correction method. The statistical information is the significance of the difference between the groups by using the t test in GraphPad Prism 9.0. The experimental data are expressed as the mean ± mean standard error (SEM).

## 3 Results

### 3.1 Allicin attenuated blood glucose in T2DM rats

**Figure 1:**
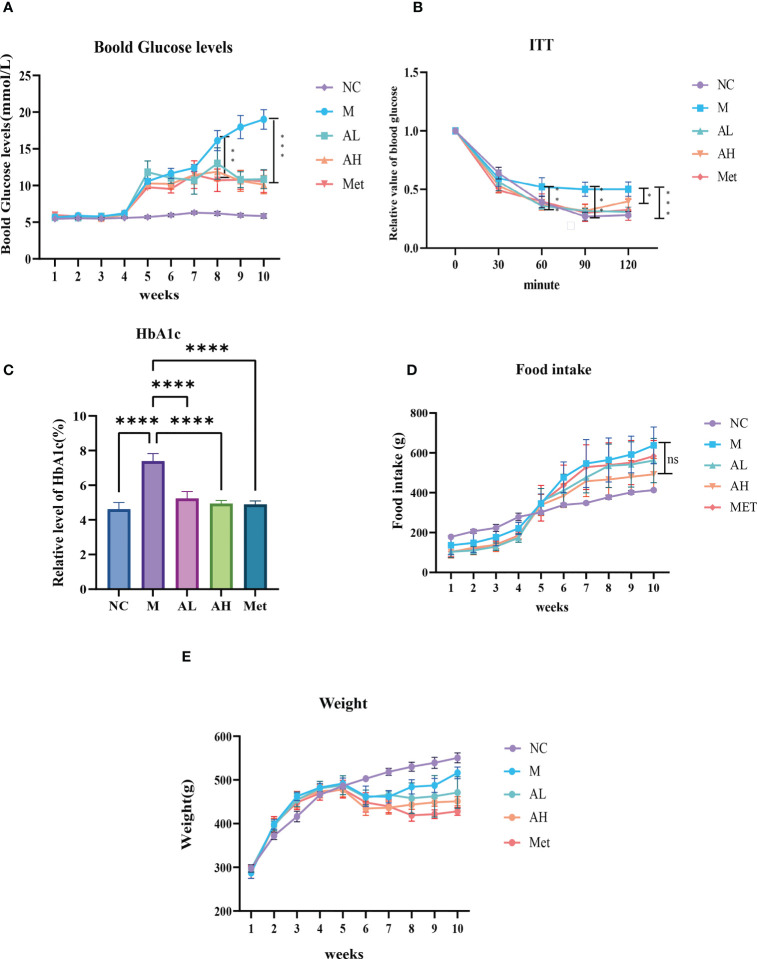
**(A)** Blood glucose levels in each group of model rats, starting from the fourth week of modeling. **(B)** At the 10th week, the effects of allicin on the alleviation of insulin resistance in rats after modeling. **(C)** Hba1c level at week 10. **(D)** Food intake information in each group of model rats at each week. **(E)** Body weight information in each group of model rats at each week. “*” Significant difference between the model group and drug group. (****p < 0.0001, ***p < 0.001, ** p < 0.01, and * p < 0.05) “ns” means there is no significant difference, n=5 for each group.

As described in the Materials and Methods, we raised five groups of rats: NC group, M group, AL group, AH group and Met group. Compared with the NC group, the blood glucose of rats in the four diabetic model groups increased one week after intraperitoneal injection of STZ, with all blood glucose values >8.4 mmol/L. According to the modeling method, the established model simulates the model of slightly higher blood glucose in patients with T2DM. Blood glucose slightly increased, but there was a significant difference from the NC group. After the fifth week, the four diabetes model groups were treated with carboxymethylcellulose sodium (15 ml/kg), low-dose allicin (30 mg/kg), high-dose allicin (70 mg/kg) and metformin (100 mg/kg). Weekly physical indicator monitoring showed that the blood glucose continued to rise in Group M, while the low-dose allicin (AL group) and high-dose allicin (AH group) gavage could significantly attenuate the rise in blood glucose beginning at week 8, which showed a similar effect with that of metformin (Met group) (**Figure 1A**, **Table 1**). At the 8th week, the high-dose allicin group and metformin group showed significant differences from the M group, while the low-dose allicin group and the M group had significant differences at the 9th week. Until the end of the experiment (the 10th week), the M group showed very significant differences from the other groups. Insulin resistance is one of the main causes of elevated blood glucose, and studies have shown that allicin can alleviate insulin resistance. Therefore, before the end of the experiment, insulin resistance tolerance was measured in each group of rats, and the normalization method was used for statistics, as shown in **Figure 1B**. The sensitivity to insulin in the allicin group was significantly greater than that in the model group at each time point from 60 min to 120 min. At the same time, HbA1c in the tenth week also showed significant differences (**Figure 1C**). These results indicate that allicin may reduce the extent of diabetic insulin resistance.

**Table 1:** Blood glucose monitoring of diseased rats after administration.

	M	AL	AH	Met	NC
Week 5(Administration)	10.58 ± 0.81	11.82 ± 3.38	10.28 ± 0.77	9.76 ± 0.40	5.70 ± 0.42^##^
Week 6	11.66 ± 1.53	11.04 ± 1.65	10.22 ± 1.6	9.54 ± 1.23	5.96 ± 0.48^###^
Week 7	12.40 ± 1.00	10.72 ± 4.23	11.44 ± 2.72	11.48 ± 4.22	6.30 ± 0.45^###^
Week 8	16.12 ± 3.06	13.00 ± 4.79	11.88 ± 2.82^**^	10.72 ± 3.41^**^	6.20 ± 0.60^###^
Week 9	17.96 ± 3.50	10.80 ± 2.42^***^	10.64 ± 3.19^***^	10.78 ± 2.89^***^	6.40 ± 0.31###
Week 10	19.00 ± 2.94	10.86 ± 2.90^***^	10.12 ± 2.77^***^	10.58 ± 3.40^***^	6.64 ± 0.49^###^

“#” Significant difference between the model group and NC group. “*” Significant difference between the model group and drug group. (###p< 0.001,## p < 0.01, # p < 0.05, ***p< 0.001,** p < 0.01, and * p < 0.05), n=5 for each group.

**Figure 2:**
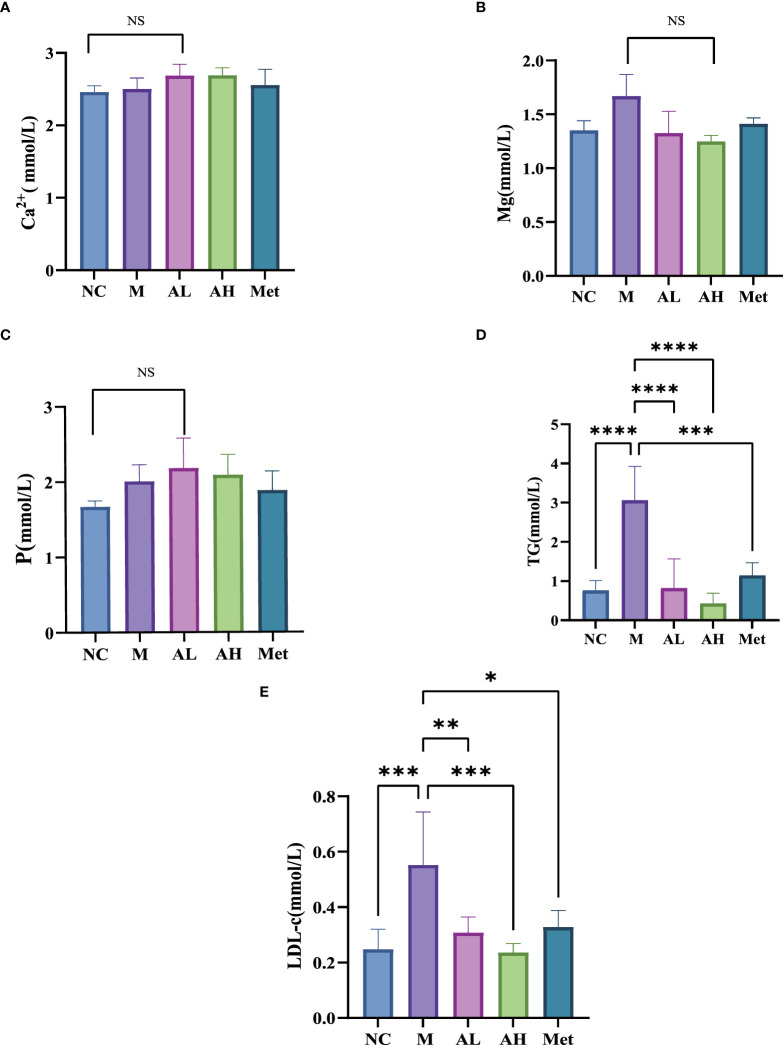
**(A–C)** Serum levels of Ca2+, P and Mg2+ in each group at week 10. **(D, E)** Comparison of TG and LDL blood lipids in each group at week 10. (****p< 0.0001, ***p< 0.001, ** p < 0.01, and * p < 0.05), “Ns” means there is no significant difference, n=5 for each group at the 10^th^ week.

The body weights of the five groups were evaluated each week and showed that from week 6 on, the weight gain of the AL, AH and Met groups remained unchanged, while that of the M group was restored to that of the NC group (**Figure 1D**). The food intake comparison showed no significant difference between the M group and the other three model groups (**Figure 1E**). Serum Ca^2+^, P and Mg^2+^ analysis showed no differences among the five groups (**Figures 2A–C**). At the same time, we tested TGs and LDL and found that, similar to metformin, allicin could also reduce serum TGs and LDL (**Figures 2D, E**). The above results indicate that allicin can regulate the weight gain, blood glucose, insulin resistance and TG/LDL increase caused by a high-fat diet. In terms of these abilities, allicin can achieve similar efficacy to the first-line drug metformin.

### 3.2 Allicin regulated the intestinal microbiota in T2DM rats

**Figure 3:**
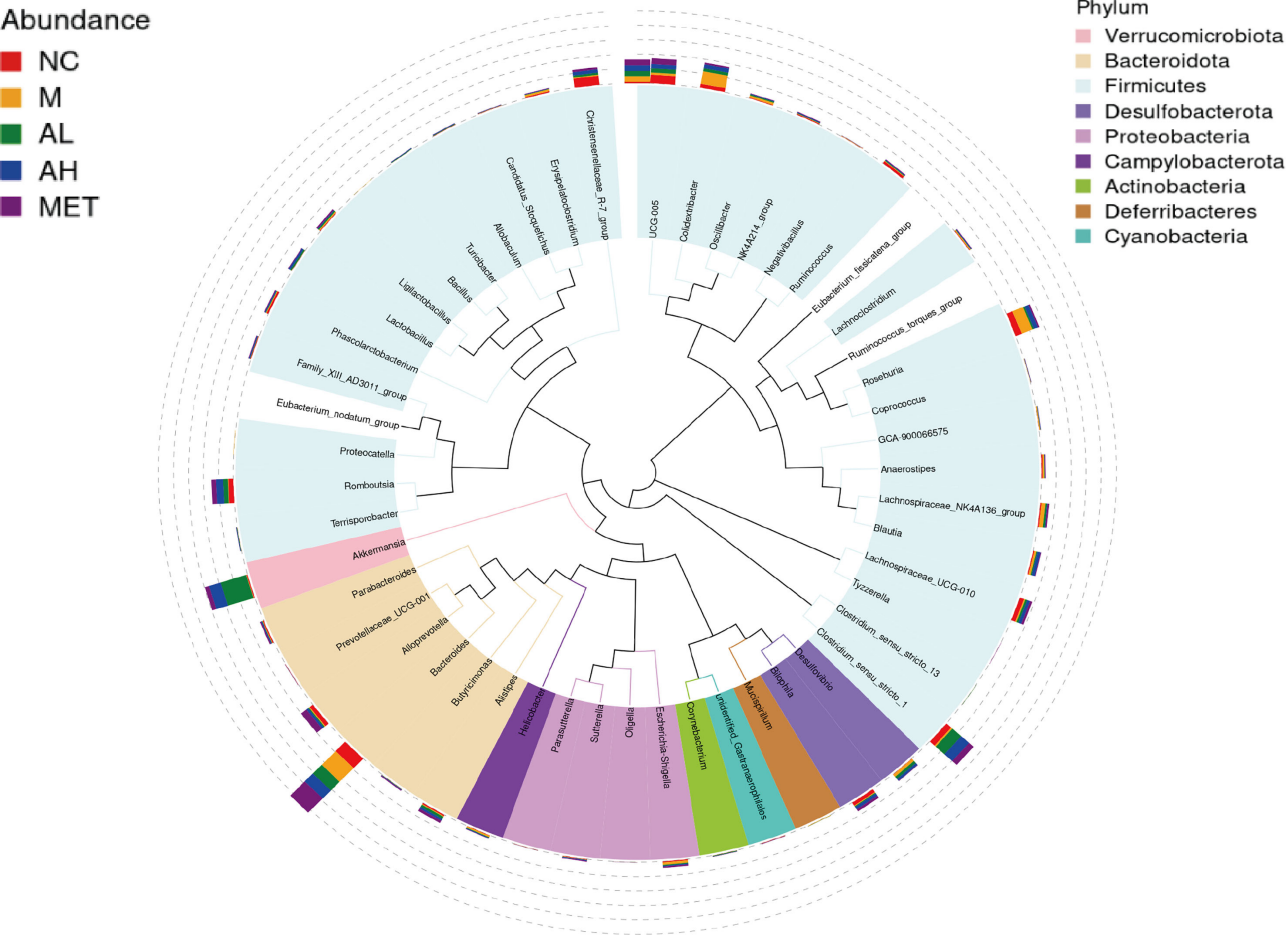
Evolutionary trees at the genus level for different groups at the same time point.

To investigate the effect of allicin on the intestinal microbiota in T2DM rats, fecal samples from the five groups were analyzed as described in the Materials and Methods. These feces were collected at the time of rat execution at week 10. The data presented in the study are deposited in the GenBank (NCBI) repository, accession number PRJNA904076. The top 50 genera were obtained by multiple sequence alignment, and a stacked histogram was drawn on the evolutionary tree (**Figure 3**).

**Figure 4:**
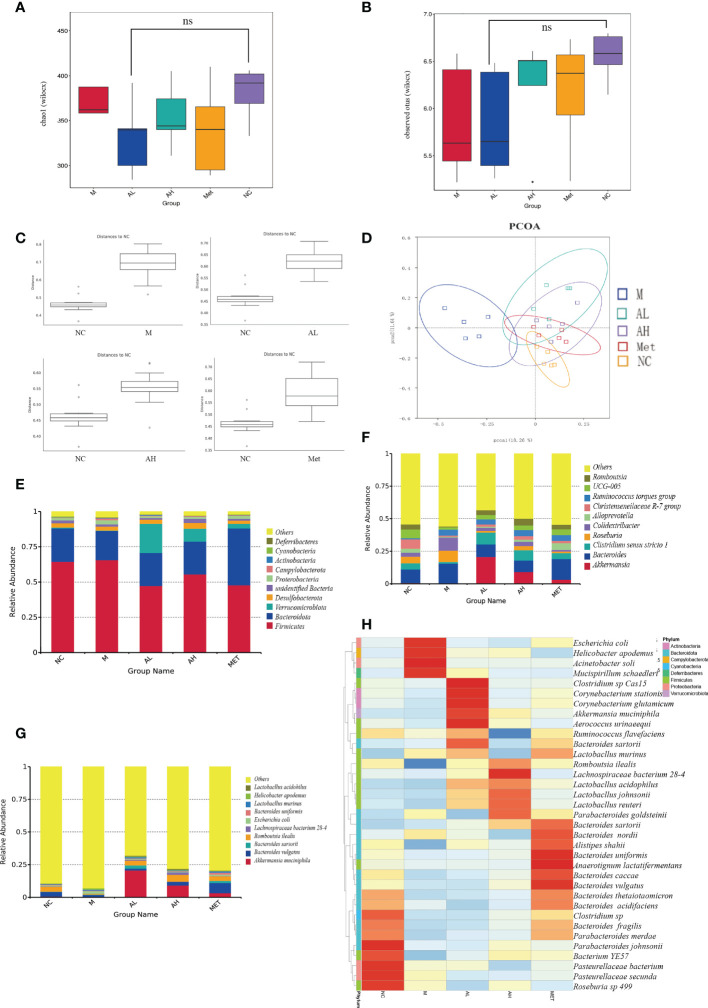
Characteristics of the intestinal microbiota among different groups of T2DM rats. **(A)** Chao1 obtained by alpha analysis. **(B)** The Observed species index obtained by alpha analysis. **(C)** Bray-Curtis distance of the fecal microbiota **(D)**. PCoA of the fecal microbiota. **(E)** The top ten microbial populations in each group at the order level. **(F)** The top ten microbial populations in each group at the genus level. **(G)** The top ten microbial populations in each group at the species level. **(H)** Heatmap of fecal microorganisms in different groups at the same time point. (* p < 0.05, ns means no significant difference), n=5 for each group, at the 10^th^ week.

Analysis of the abundance and structure of intestinal contents of five groups of models at the species level. For alpha diversity, the observed species index and Chao1 analysis showed that allicin did not significantly reduce the abundance of intestinal contents after administration (**Figures 4A, B**). However, in addition, based on the NC group, we tested the significance of the differences in the community structure of the rats in the intervention group. The ANOSIM test showed that the deviation level of the M group was the highest, with an R value of 0.732 (p=0.039), AL group R=0.692 (p=0.0199), AH group R=0.635 (p=0.014), and Met group R=0.364 (p=0.0149) (**Figure 4C**). We found that the intestinal microflora in group M changed the most, while the differences in the administration group all decreased. Principal coordinate analysis (PCoA) showed that the AL and AH groups shared more identity with the NC group than the M group (**Figure 4D**). The above results indicate that allicin could influence the overall characteristics of the intestinal microbiota.

The main microbial composition analysis showed that the top 10 phyla of intestinal microbes were similar among the five different groups, while allicin gavage increased the relative abundance of Verrucomicrobiota (**Figure 4E**). At the genus level, allicin and metformin gavage increased the abundances of Akkermania and Clostridium, while the M group harbored more genera of Colidextribacter (**Figure 4F**). At the species level, *Akkermansia muciniphila* was dramatically increased in the allicin and metformin gavage groups (**Figure 4G**). The first 35 species with total abundance at the classification level were mapped into a heatmap (the bacterial abundance was the average abundance in all samples within each group) (**Figure 4H**). As shown in the heatmap, the positive and negative values represent the relative content of the flora. The darker the red color is, the greater the relative content is, and the closer it is to 1. The blue is the opposite. The allicin gavage groups (AL and AH groups) were dominated by probiotic bacteria, such as *Akkermansia muciniphila*, *Aerococcus urinaeequi*, *Lactobacillus murinus*, *Romboutsia ilealis*, *Lachnospiraceae bacterium 28-4*, *Lactobacillus acidophilus*, *Lactobacillus johnsonii*, and *Lactobacillus reuteri*, while the M group was more enriched with conditionally pathogenic bacteria, such as *Escherichia coli*, *Mucispirillum schaedleri* and *Helicobacter_apodemus*. Studies have reported that *Mucispirillum schaedleri* induces inflammation in the intestinal environment and increases the severity of inflammatory bowel disease, systemic inflammation and T2DM (Herp et al., 2021). *Helicobacter pylori* can induce damage to the gastric mucosa to increase inflammation and insulin resistance (Ojetti et al., 2010). In contrast, allicin gavage-enriched bacteria, such as *Akkermansia muciniphila* and *Lactobacillus* species, were reported to act as probiotics in the treatment of T2DM (Zhang L. et al., 2018). The beneficial bacteria enriched in the intestinal flora after administration of allicin were significantly higher than those after administration of metformin, which indicates that allicin is superior to metformin in changing the structure of the intestinal flora.

**Figure 5:**
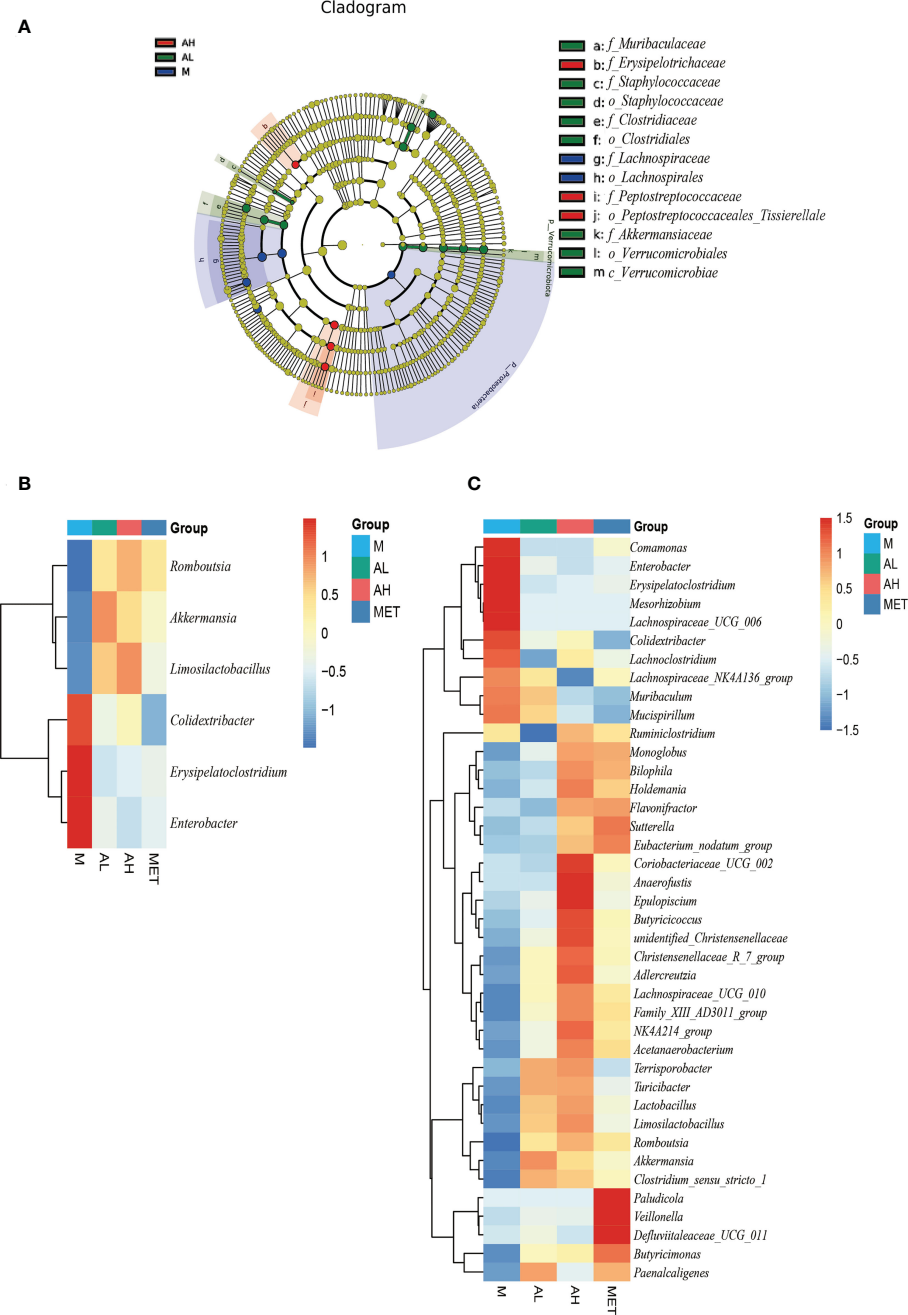
Analysis of four groups with significantly different intestinal microbiota at the 10^th^ week. **(A)** Difference analysis of LEfSe (LDA effect size, used to compare the statistical significance and correlation of the three groups of bacteria) with the threshold set to 4. **(B)** The significant differences among the four groups of rats were analyzed by heatmap, and the intersection of sets was taken. **(C)** The significant difference of the four groups of rats was analyzed by heatmap, union of set (The significance is P<0.05).

LEfSe analysis also confirmed this point. Through LDA analysis with a threshold of 4, we obtained a relatively strict significance analysis difference diagram between the M group, AL group and AH group (**Figure 5A**). Beneficial species such as Akkermansia and Clostridiales in the allicin group increased significantly, while conditional pathogenic bacteria such as Erysipelotrichaceae were still enriched in the M group. Next, we carried out Wilcox analysis at the genus level between the M and the AL, AH, and Met groups and integrated the three difference analysis results into **Figures 5B, C**. *Romboutsia*, *Akkermansia*, and *Limosilactobacillus* were significantly increased in the allicin gavage groups, while *Colidextribacter*, *Erysipelatoclostridium*, and *Enterobacter* were enriched in the M group. Moreover, compared to the M group, the AL group exhibited significant enrichment of the *Christensenellaceae* R7 group, *Adlercreutzia*, *Lachnospiraceae* UCG 010, *Acetanaerobacterium*, *Terrisporobacter*, *Turicibacter*, *Lactobacillus*, *Limosilactobacillus*, *Romboutsia*, *Akkermansia*, and *Clostridium sensu stricto*. In addition to these microbes, the AH group was also enriched with more *Ruminiclostridium*, *Monoglobus*, *Bilophila*, *Holdemania*, *Flavonifractor*, *Sutterella*, *Eubacterium nodatum* group, *Coriobacteriaceae* UCG 002, *Anaerofustis*, *Epulopiscium*, *Butyricicoccus*, and *unidentified Christensenellaceae* than the AL group. The above results show that allicin gavage can significantly increase the abundance of a variety of probiotics, such as *Lactobacillus*, *Limosilactobacillu*, *Romboutsia*, *Akkermansia*, and *Clostridium*. In contrast, opportunistic pathogens, such as *Comamonas*, *Enterobacter*, *Erysipelatoclostridium*, *Mesorhizobium*, *Lachnospiraceae UCG 006*, *Colidextribacter*, *Lachnoclostridium*, and *Lachnospiraceae NK4A136 group*, were more enriched in the M group. The above results indicate that the prebiotic effect of allicin on blood glucose control in T2DM rats may occur through modulating the intestinal microbiota.

### 3.3 Allicin increased intestinal SCFA levels and decreased the serum inflammatory index

**Figure 6:**
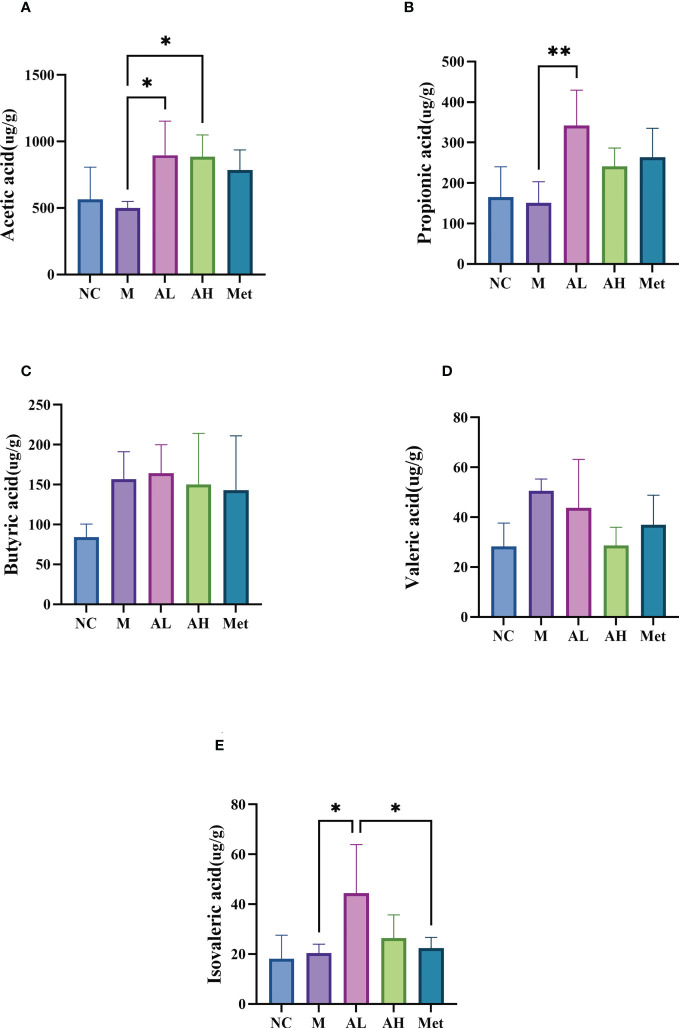
SCFAs in the colon contents. **(A)** Acetic acid, **(B)** propionic acid, **(C)** butyric acid, **(D)** valeric acid, and **(E)** isovaleric acid. (** p < 0.01, and * p < 0.05), n=5 for each group at the 10^th^ week.

To test whether allicin gavage could increase beneficial metabolites in the intestine, we analyzed five SCFAs: acetic acid, propionic acid, butyric acid, valeric acid and isovaleric acid. Compared with the M group, acetic acid, propionic acid and isovaleric acid were significantly increased in the AL group, and acetic acid was also significantly increased in the AH group (**Figure 6A**). SCFAs are produced by the intestinal microbiota and were reported to have a prebiotic effect on T2DM in a previous study (Canfora et al., 2019). Enrichment of acetic acid can slow weight gain in T2DM rats with glucose tolerance (Yamashita, 2016). These results suggest that allicin plays a role in relieving the development of diabetes by modulating the intestinal microbiota and promoting the production of beneficial intestinal metabolites, such as SCFAs (**Figures 6B–E**).

**Figure 7:**
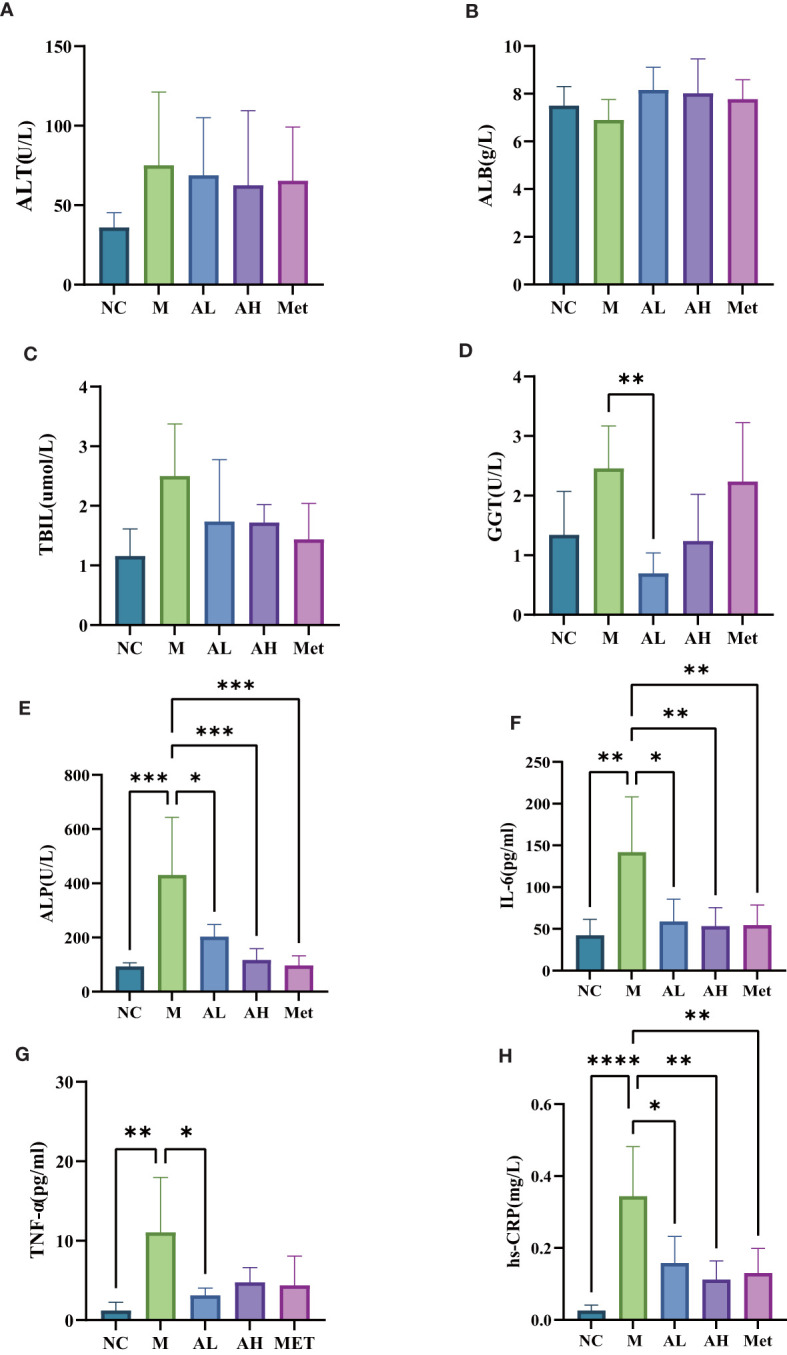
Serum levels of **(A)** alanine aminotransferase, **(B)** albumin, **(C)** total bilirubin, **(D)** glutamyl transpeptidase, **(E)** alkaline phosphatase, **(F)** interleukin-6, **(G)** tumor necrosis factor TNF-α, and **(H)** C-reactive protein among five different groups of rats. (****p< 0.0001, ***p< 0.001, ** p < 0.01, and * p < 0.05), n=5 for each group at the 10^th^ week.

To investigate the prebiotic effects of allicin gavage on T2DM rats, we further analyzed the serum biochemical indexes and inflammatory factors that are potentially related to the alteration in the intestinal microbiota induced by allicin. Of the liver function-related indexes, combined with bilirubin measurements, allicin gavage showed no acute toxic effect on the liver in T2DM rats (**Figures 7A–C**), while allicin gavage, especially in the low-dose group, significantly reduced the levels of serum ALP and GGT (**Figures 7D, E**), which suggests that allicin has a protective effect on chronic liver injury. As the serum inflammatory index increase is one of the performance indicators for the development of T2DM, we examined the serum inflammatory factors hs-CRP, IL-6 and TNF-a and found that compared with the M group, the levels of hs-CRP and IL-6 in the AL and AH groups were significantly lower, and the level of TNF-a in the AL group was also significantly different from that in the M group (**Figures 7F–H**), which suggests that allicin can alleviate the systemic inflammation caused by T2DM and has no side effects.

### 3.4 Correlation analysis between intestinal bacteria and clinical indicators

To explore the connection between the intestinal microbiota and clinical index parameters, we conducted Spearman correlation analysis between these two datasets.

**Figure 8:**
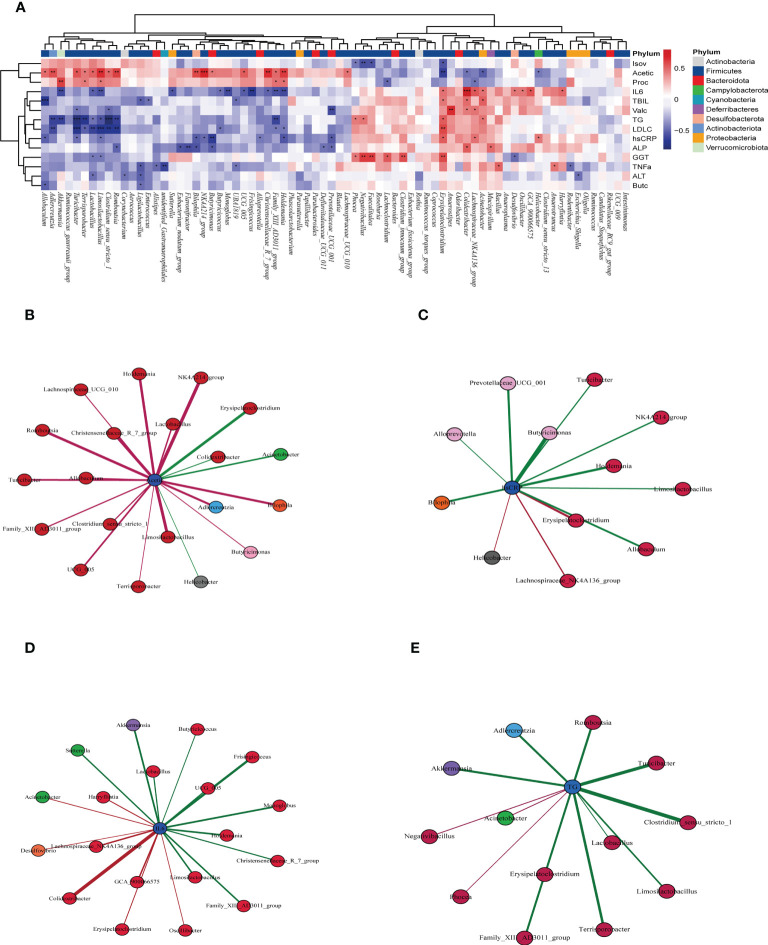
**(A)** Correlation of the intestinal flora with clinical indicators by Spearman correlation analysis (red indicates a positive correlation, blue indicates a negative correlation, and * indicates a significant difference). “*” in red indicates that the bacteria had a significant enrichment effect on the clinical index, and “*” in blue indicates that the bacteria had a significant reduction effect on the clinical index ***p < 0001, ** p < 0.01, and * p < 0.05, n=5 for each group, at the 10^th^ week.), **(B)** microbiota closely related to acetic acid, **(C)** microbiota closely related to hs-CRP, **(D)** microbiota closely related to IL-6, **(E)** and microbiota closely related to TG (the significance of B-E was P<0.05).

We found that substantial intestinal microbes were closely correlated with clinical indicators. The increase in intestinal microbes by allicin gavage was positively correlated with various health indicators but negatively correlated with indicators of T2DM. For example, *Akkermansia* was positively correlated with propionic acid and negatively correlated with IL-6 and TGs; *Limosilactobacilus* was positively correlated with acetic acid and propionic acid and negatively correlated with IL-6, LDL-C and hs-CRP; and *Romboutasia* was positively correlated with acetic acid and negatively correlated with TGs, LDL-C, and ALP (**Figure 8A**). However, the dominant strains in group M, such as Colidextribacter, showed a negative correlation with acetic acid and a positive correlation with IL-6, TBIL, and ALP. Erysipelatoclostridium showed a negative correlation with acetic acid and isovaleric acid and a positive correlation with TGs, LDL-C, hs-CRP, IL-6, and GGT (**Figure 8A**). The above results indicate that the changes in the intestinal microbiota induced by allicin gavage are closely related to the improvement in T2DM symptoms.

Afterward, we carried out correlation analyses between four of the important indicators and the intestinal microbes. Acetic acid was positively correlated with allicin gavage-increased microbes, such as *Allobaculum*, *Clostridium*_sensu_stricto_1, *Limosilactobacillus*, *Romboutsia*, and *Lactobacillus*, while it was negatively correlated with microbes enriched in the M group, such as *Erysipelatoclostridium*, *Colidextribacter* and *Helicobacter* (**Figure 8B**). The microbes positively related to hs-CRP, IL-6, and TGs were mainly from group M, while the microbes negatively related to those factors were more likely to come from the allicin gavage group (**Figures 8C–E**).

## 4 Discussion

The gut microbiota is of great importance to the digestion/supply of nutrients, maintenance of gut barrier function, development/modulation of the immune system and production of a group of prebiotic metabolites, such as SCFAs (Chassard and Lacroix, 2013; Jayashree et al., 2014; Ratajczak et al., 2019). Dysbiosis of the intestinal microbiota is closely related to the development of metabolic disorders, and intestinal microbiota-targeted therapeutics have great promise in the treatment of metabolic diseases, such as T2DM, obesity, gout, malnutrition and osteoporosis (Guo et al., 2016; Marchesi et al., 2016; Fluitman et al., 2017; Xu et al., 2017; Fassarella et al., 2021). In this study, we constructed a T2DM rat model, treated the rats with allicin by gavage, and compared the clinical parameters among the five groups to verify the prebiotic effects of allicin on T2DM. The results showed that allicin can modulate the intestinal microbiota, reduce the serum inflammatory index, and increase the abundance of intestinal SCFAs. In addition, allicin gavage reduced TG and LDL levels and alleviated chronic liver injury. In general, allicin has a prebiotic effect on alleviating blood glucose, inflammation and other metabolic abnormalities.

Previous studies have shown the effect of allicin on hypolipidemia (El-Sheakh et al., 2016; Shi et al., 2019). Our study, which was an allicin intervention experiment specifically conducted for T2DM, confirmed that allicin has an ideal prebiotic effect on blood glucose and showed that low-dose allicin gavage may have a better effect. Furthermore, allicin gavage did not cause acute liver injury, which is in agreement with previous studies (Wang et al., 2015; Chen et al., 2018; Samra et al., 2020). Our study also suggests that allicin gavage may have a prebiotic effect on chronic liver injury, while the specific mechanisms related to allicin treatment need to be further investigated.

Our study showed that low-dose gavage of allicin could dramatically improve the abundance of *Akkermansia muciniphila* and *Lactobacillus*. *Akkermansia muciniphila* has been reported to improve T2DM by reducing gluco/lipotoxicity, oxidative stress and inflammation damage and normalizing the intestinal microbiota in streptozotocin-induced diabetic rats (Zhang L. et al., 2018; Shih et al., 2020; Wang et al., 2020). *Lactobacillus* has also been proven by multiple studies to regulate metabolic abnormalities (Chen et al., 2018), including the regulation of blood glucose and lipid metabolism and the improvement in oxidative stress and the inflammatory response (Yan et al., 2019). Previous studies showed that intestinal *Lactobacillus acidophilus* was significantly reduced in diabetic patients compared to healthy controls (Halawa et al., 2019), while allicin could recover the abundance of the genus *Lactobacillus*.

Allicin may also inhibit the abundance of opportunistic pathogens in the intestine. In our results, of the top 35 most abundant species, *Helicobacter apodemus, Mucispirillum schaedleri, Micromonospora echinospora*, etc., were enriched in the M group. *Helicobacter apodemus* was reported to be associated with a rise in systemic inflammation (Kim et al., 2018), *Mucispirillum schaedleri* could increase intestinal inflammation (Herp et al., 2021), and *Micromonospora echinospora* was toxic to liver (Hep G2 cells) cells (Fang et al., 2020).

In addition, our study showed that allicin gavage dramatically improved the abundance of SCFAs. SCFAs are prebiotic metabolites produced by the intestinal flora (Canfora et al., 2019) and are reported to control the development of diabetes through a number of mechanisms (Yap and Mariño, 2021). For example, acetic acid could affect parasympathetic activity through microbial-nutritional interactions, increase gastric hunger hormone, stimulate insulin secretion, and improve nutrient utilization (Yamashita, 2016). Propionic acid also showed an antidiabetic effect (Cai et al., 2006). However, a few studies have reported that isovaleric acid is related to T2DM, and a study showed its correlation with diabetic ketoacidosis (Kılıç et al., 2014).

In this experiment, we compared the effects of allicin in the two dose groups. Interestingly, we found that the effect of the low-dose allicin group seemed to be superior to that of the high-dose allicin group in some respects. Based on previous studies, we believe that this may be caused by the strong bactericidal effect of allicin (Ankri and Mirelman, 1999; El-Saber Batiha et al., 2020). In the β analysis, we can see that there was no significant difference between the high-dose allicin and NC groups, while the M group was the group with the largest change in flora, and there was also a significant difference between the M and AL groups. The reason for the change in the AL group flora structure may still be in the process of treating T2DM through flora, so there are more therapeutic bacteria. At the same time, the result of enriching too many beneficial bacteria is to enrich some metabolites, which provides the best representation of the therapeutic effect.

Compared with traditional drugs for T2DM, the performance of allicin is surprising. In the experiment, we can clearly see that metformin has similar effects to allicin in controlling blood glucose and reducing body fat. In addition, metformin is now a fairly mature first-line drug, and allicin seems to have the potential to surpass metformin, for example, in regulating the structure of flora and enriching the metabolic products of intestinal flora. According to this experiment, allicin is the same as metformin in improving the apparent symptoms of diabetes, but in the long run, the prebiotic ability of allicin on intestinal flora cannot be ignored. However, the side effects of metformin have been commonly reported (de Jager et al., 2010; DeFronzo et al., 2016; Wang and Hoyte, 2019), such as lactic acid poisoning, vitamin B-12 deficiency, gastrointestinal discomfort and other risks. Allicin as a prebiotic is currently very safe due to its mild efficacy. Similar prebiotics have also been mentioned in previous studies. Prebiotics such as berberine and inulin can also enrich intestinal metabolites and reduce the level of inflammation (Pan et al., 2019). However, the therapeutic effect of prebiotics, including traditional prebiotics, is not as good as that of allicin. For example, berberine has difficulty controlling fasting blood glucose, blood lipids and liver enzymes (Nejati et al., 2022). In addition, it has been reported that prebiotics such as berberine reduce the intake of diet in the model group (Zhang et al., 2019), so whether this variable will lead to blood glucose differences remains to be further studied.

As one of the most widely grown ingredients in the world, allicin has good bioavailability and bioequivalence (Lawson and Hunsaker, 2018), which makes it easy for people to obtain allicin compared with other probiotics with strict requirements. In fact, allicin is widely used in the clinic for cancer, cardiovascular disease, cold, hypertension, etc (Majewski, 2014; Sarvizadeh et al., 2021; Zhou et al., 2022). This article established the relationship between allicin and the improvement of diabetes through the intestinal flora and provided a new idea for human beings to use allicin and improve diabetes. Moreover, compared with traditional prebiotics, allicin is still in its infancy in the prevention and treatment of diabetes, and there are still a large number of pathways and mechanisms that are unknown, which makes the potential of allicin very great. In the future, we can integrate various prebiotics and combine their advantages to provide new research ideas for the prevention and treatment of diabetes.

In conclusion, our results showed that allicin can modulate the intestinal microbiota and regulate the metabolites produced by intestinal microbes and is a potential new drug for the treatment of T2DM.

## Data availability statement

The data presented in the study are deposited in the GenBank (NCBI) repository, accession number PRJNA904076.

## Ethics statement

The animal study was reviewed and approved by Endocrine and Metabolic Disease Hospital, Shandong First Medical University, 2021-005.

## Author contributions

XZ and WD designed the research, LL and YY conducted the animal model study, XW, YH, LD, ZW, and QS wrote and revised the manuscript. All authors contributed to the article and approved the submitted version.
